# Response of Vegetation Greenness to Extreme Droughts and Possible Mechanisms in Guizhou Province, China

**DOI:** 10.1002/ece3.72945

**Published:** 2026-01-25

**Authors:** Chuncan Meng, Yingqing Cen, Xu Xue

**Affiliations:** ^1^ Department of Ecology, College of Life Science Guizhou University Guiyang Guizhou Province China; ^2^ Key Laboratory of Plant Resource Conservation and Germplasm Innovation in Mountainous Region (Ministry of Education), Collaborative Innovation Center for Mountain Ecology & Agro‐Bioengineering (CICMEAB), College of Life Science/Institute of Agro‐Bioengineering Guizhou University Guiyang Guizhou Province China

**Keywords:** drought, Guizhou Province, hydrothermal condition, karst and non‐karst landforms, NDVI, vegetation response

## Abstract

Guizhou Province, China, experienced two extreme drought events during the periods of autumn to spring 2009–2010 and spring to autumn 2011. These events, classified as “one‐century drought,” led to severe water shortages, significant declines in crop yield, and substantial socioeconomic losses. Despite the evident impact, the heterogeneity of vegetation responses, measured using the Normalized Difference Vegetation Index (NDVI), across different vegetation types and landforms in this region remains insufficiently quantified. Therefore, this study investigates the spatiotemporal evolution of these two events and evaluates the differential NDVI responses of vegetation using multiple climatic datasets and remote sensing‐derived vegetation indices. Results indicate that both drought events reached severe levels and persisted for approximately 8 months. The southwestern and western parts of Guizhou Province were hit the hardest. The 2009–2010 drought exerted a more pronounced inhibitory effect on vegetation growth, as evidenced by significantly greater reductions in NDVI during the spring season. Analysis across five major vegetation types showed universal declines in NDVI anomalies, with meadows experiencing the most severe suppression, followed by scrublands, broadleaf forests, and grasslands. Needleleaf forests exhibited the highest drought tolerance. In contrast, the 2011 drought had a comparatively milder impact on vegetation dynamics. Notably, certain vegetation types, particularly broadleaf forests, displayed sustained greening even under drought conditions. Furthermore, vegetation growing on karst landforms exhibited more pronounced declines in greenness compared to those on non‐karst landforms, highlighting the influence of geological substrates on drought sensitivity. These findings suggest that under conditions of extreme drought severity, alterations in hydrothermal conditions significantly affect vegetation response patterns across both vegetation types and landform categories. This study provides important insights into the ecological impacts of drought in karst‐dominated regions and offers a scientific basis for formulating drought risk management strategies and ecological conservation policies in Guizhou Province.

## Graphical abstract

Spatiotemporal variations of the two drought events and the different response of vegetation NDVI to both events were investigated based on multiple data in Guizhou Province. The 2009–2010 drought exerted a inhibitory effect on vegetation growth, while the 2011 drought exhibited a milder impact, even demonstrating continued green growth amidst the dry spell. Under drought conditions, alterations in hydrothermal conditions could influence the response dynamics of vegetation across various vegetation types and both types of landscapes.

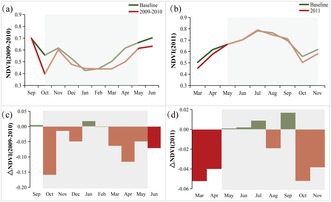

## 1 Introduction

Drought is a complex and highly destructive natural hazard, characterized by a prolonged deficiency in precipitation that significantly deviates from long‐term climatic averages. This hydrometeorological imbalance leads to a sustained mismatch between water availability and demand, with profound and wide‐ranging impacts on ecological systems and socio‐economic structures (Dai 2011; Vicente‐Serrano et al. 2013; Cunha et al. 2019). Climate change, particularly global warming, has intensified the severity, duration, and frequency of drought events, signaling the potential for widespread impacts at global, regional, and local scales (IPCC 2013, 2021; Shi et al. 2017; Judi et al. 2018). Extensive research has highlighted the detrimental effects of drought on vegetation growth, including the exacerbation of forest fires, degradation of ecosystem health, disruption of the carbon balance, and ultimately, the deterioration of entire ecosystems (Zhang et al. 2012; Xu, Wang, et al. 2021; Cao et al. 2022). Accurate estimation of drought frequency, intensity, duration, and the extent of its environmental influence is critical for improving ecological protection strategies and strengthening disaster risk management. The Standard Precipitation Evapotranspiration Index (SPEI; Vicente‐Serrano et al. 2010), which integrates both temperature and precipitation data, has emerged as a widely adopted metric for drought monitoring by incorporating the advantages of both the Palmer Drought Severity Index (PDSI; Palmer 1965) and the Standardized Precipitation Index (SPI; McKee et al. 1993).

Vegetation serves as a critical component of terrestrial ecosystems, playing a pivotal role in regulating land‐atmospheric energy exchanges, the global cycling of matter and energy, and overall ecosystem stability (Hou et al. 2015; Chen et al. 2021; Xue et al. 2023). The future trajectory of these ecosystem functions depends heavily on vegetation responses to drought conditions (Humphrey et al. 2018; Miguez‐Macho and Fan 2021). However, the response of terrestrial vegetation to drought is inherently complex, governed by multiple factors. These include the timing, intensity, duration, and spatial extent of drought events, as well as ecosystem‐specific characteristics such as vegetation type and structural composition (Wu and Chen 2013; Tang and Dubayah 2017; Swann 2018; Jin et al. 2023). Prolonged drought can induce widespread vegetation mortality, triggering substantial structural reorganization and functional degradation within terrestrial ecosystems (Zhou, Wang, et al. 2018; Shao et al. 2022). Such disruptions may result in significant losses of biomass and carbon stocks, ultimately contributing to severe ecosystem degradation and the emergence of ecological crises (Zhao and Running 2010; Zhang et al. 2012; Zhou, Luo, et al. 2018). Consequently, understanding the spatiotemporal dynamics of drought events and their associated impacts on vegetation has become a central focus in ecological and environmental research (Li et al. 2019; Liu, Chen, et al. 2021).

Currently, both domestic and international researchers have made significant contributions to understanding vegetation responses to drought. However, drought events exhibit pronounced spatial heterogeneity, with considerable variation in their intensity, duration, and ecological impacts across regions characterized by differing climatic conditions and geomorphic landforms (Xu, Wang, et al. 2021). Recent studies have further demonstrated that vegetation responses to drought vary significantly among different ecosystem types, largely due to differences in morphological traits and physiological adaptations among vegetation types (Ding et al. 2020; Wu, Zhong, et al. 2024). In Iran, Fathi‐Taperasht et al. (2022) identified distinct drought response patterns among vegetation types, noting that scrublands and croplands experienced more prolonged drought impacts and exhibited slower recovery rates compared to forest areas. Similarly, Wu, Yin, et al. (2024) reported that in the upper West River Basin (southwestern region), drought conditions were both more severe and of longer duration. In the context of the 2009–2010 drought in southwestern China, Zhao et al. (2015) observed early signs of vegetation suppression in grassland and woody savanna, indicating a heightened sensitivity to initial water stress. Notably, although drought conditions typically suppress vegetation growth, some studies have reported enhanced forest greening and increased productivity during specific drought events. For instance, Zhang et al. (2012) and Song et al. (2019) documented increased forest productivity during the summer drought of 2011. In contrast, Dong et al. (2022), in their analysis of the 2009–2010 drought in Yunnan Province, found that forests experienced more pronounced suppression in growth compared to grassland and agricultural lands. Moreover, vegetation vulnerability and resilience to drought are significantly influenced by geomorphic features, soil properties, and prevailing climatic conditions (Huang et al. 2021; Machado‐Silva et al. 2021; Jiao et al. 2021; Xu, Wu, et al. 2021; Yao et al. 2022; Xue et al. 2024). These findings underscore the context‐dependent nature of drought‐vegetation interactions, emphasizing the critical need to quantify drought impacts on vegetation growth across different plant functional types and landform units at local scales. Such assessments are essential for informing targeted ecosystem management and conservation strategies.

In the context of ongoing climate change, the southwestern region of China has experienced a notable increase in the frequency, severity, and spatial extent of drought events (Yu et al. 2013; Qin et al. 2021; Wang et al. 2015). Guizhou Province, located in the eastern portion of Southwest China, is characterized by a highly complex topography and extensive karst landforms. These features contribute to its designation as an ecologically vulnerable region, with widespread rocky desertification and heightened environmental sensitivity (Xiao et al. 2019). The region's ecological fragility is further intensified by the impacts of climate change, posing serious challenges to environmental sustainability and resilience. Precipitation distribution in Guizhou Province is highly variable, both seasonally and spatially, primarily influenced by monsoon circulation and the region's rugged terrain. Drought is a recurrent climatic hazard, with recurrence intervals estimated at approximately 3 years for moderate events, 5 years for severe events, and 10 years for extreme events, based on historical data (Cheng et al. 2018; He et al. 2022). Despite the frequent occurrence of drought, comprehensive understanding insights into the responses of vegetation to these events in Guizhou Province remain limited. There exists a pronounced spatial variability in both drought incidence and vegetation response, which has not been sufficiently addressed in the current body of literature. Moreover, most prior studies have predominantly concentrated on the long‐term trend analysis of vegetation response to drought over extended time scales, often neglecting the heterogeneity of vegetation types and geomorphic settings within Guizhou Province. Therefore, a comprehensive investigation into the response of vegetation greenness across diverse vegetation types and geomorphic landforms to drought events is essential. Such an examination will significantly enhance the understanding necessary to inform and refine localized climate adaptation and mitigation strategies.

In the present study, we employed the SPEI over a 6‐month period (hereafter referred to as SPEI–6) to delineate two distinct drought events occurring in Guizhou Province, occurring during 2009–2010 and 2011. Utilizing the NDVI as an indicator of vegetation growth status, this study addresses the following research questions: (1) What are the spatial and temporal characteristics of the intensity, duration, and spatial extent of these two consecutive drought events across different seasons in Guizhou Province? (2) How does the NDVI respond to these extreme drought events, and what are the differential response patterns among various vegetation types and geomorphic landforms? (3) To what extent do hydrothermal conditions influence vegetation greenness during periods of extreme drought? Addressing these questions will advance the mechanistic understanding of karst ecosystem resilience, inform targeted ecological conservation efforts in southwestern China, and contribute evidence‐based strategies for effective drought risk management.

## 2 Data and Methods

### 2.1 Study Area

**FIGURE 1:**
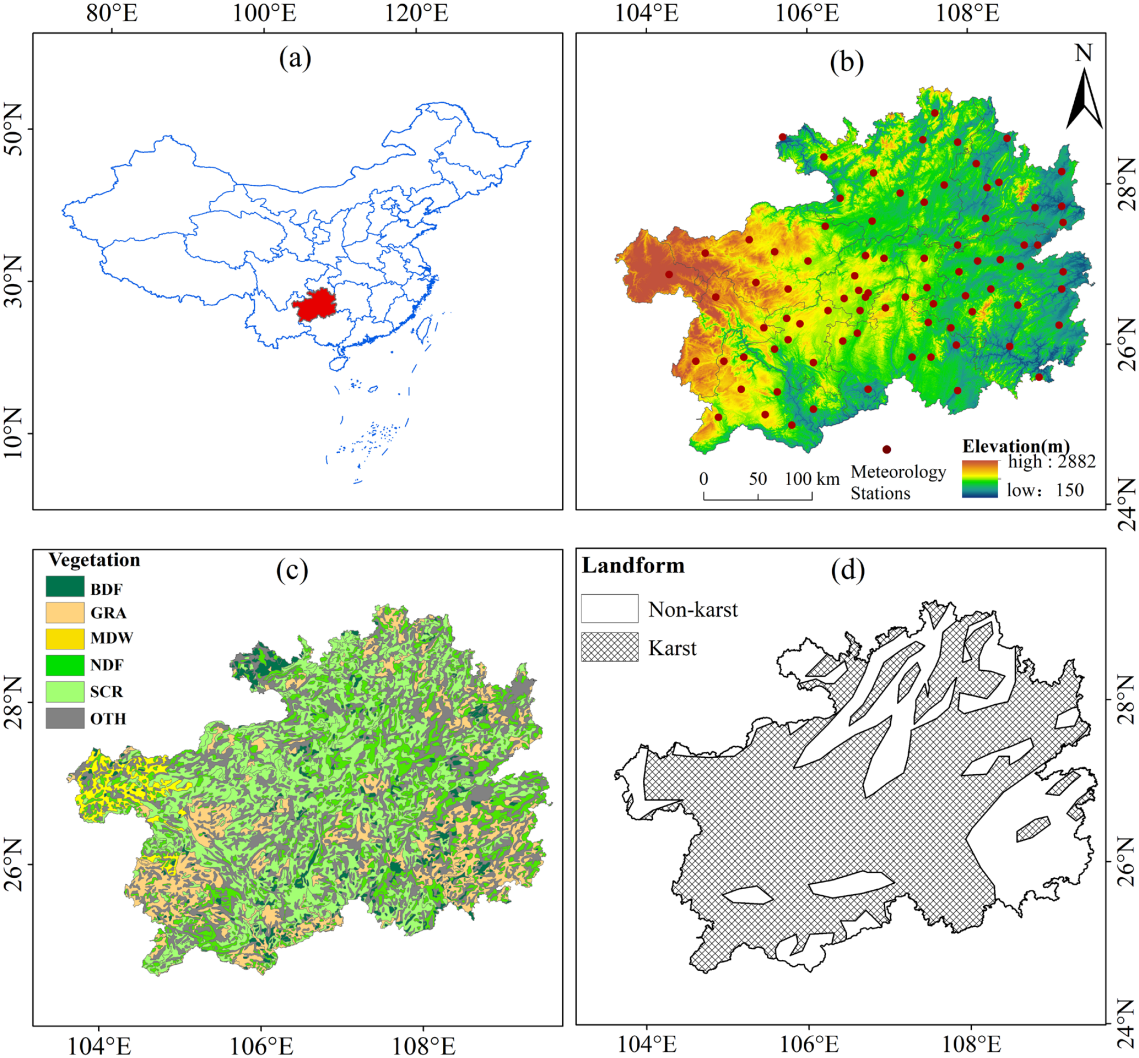
Spatial characteristics of the study area: (a) geographical location of Guizhou Province, China. (b) topography (m) and distribution of 83 national meteorological stations. (c) vegetation types, including evergreen broadleaf forests (BDF), grasslands (GRA), meadows (MDW), evergreen needleleaf forests (NDF), scrublands (SCR), and others (OTH, comprising croplands, urban areas, barren land, and wetlands). (d) classification of karst and non‐karst landforms.

Guizhou Province, strategically positioned in Southwest China (approximately 24°37′–29°13′N latitude and 103°36′–109°35′E longitude; Figure 1a), serves as a transitional zone between the hilly plains and the drainage divide of the Yangtze River and Pearl River (Chen et al. 2023). This unique geographical positioning makes Guizhou the only province in China without plains. The province's topography is characterized by a pronounced west‐high, east‐low gradient, with an average elevation of approximately 1100 m (Figure 1b). The region's complex and varied terrain gives rise to a diverse climate landscape, with significant vertical climatic variations between high mountainous plateaus and river valley terraces. This topographical and climatic diversity supports a wide range of vegetation types, including broad‐leaved forests (BDF), needleleaf forests (NDF), grasslands (GRA), meadows (MDW), and scrublands (SCR) (Figure 1c). Guizhou Province is located within the subtropical monsoon climate zone, where the mean annual temperature is approximately 15°C. The coldest month, January, typically experiences temperatures ranging from 3°C to 6°C, while the warmest month, July, records temperatures between 22°C and 25°C. The province's pronounced wet and dry seasons are significantly governed by the South Asian monsoon, with average annual precipitation ranging from 1100 to 1400 mm and exhibiting a clear north‐to‐south increasing gradient. The majority of precipitation occurs between April and October, accounting for approximately 80% of the annual total (Chen, He, et al. 2024). Moreover, as the core region of karst topography in Southwest China, Guizhou Province is predominantly covered by karst formations, which constitute approximately 73% of the landscape (Figure 1d) (Qin et al. 2021).

### 2.2 Datasets

In this study, a comprehensive dataset was employed, comprising meteorological data, normalized difference vegetation index (NDVI), land cover, geomorphological information, and digital elevation model (DEM) data. Detailed descriptions of these datasets are provided in Table 1. Daily temperature and precipitation records were obtained from national‐level meteorological stations across Guizhou Province, provided by the China Meteorological Administration. Following the compilation of monthly climatic variables, stations with more than 5% missing data were excluded, resulting in the selection of 83 representative stations. Monthly precipitation and temperature data from 2001 to 2021 were subsequently used to calculate the Standardized Precipitation Evapotranspiration Index (SPEI) dataset using R software (Figure 1b). Additionally, hydrothermal variables, including net shortwave radiation flux, surface soil moisture, and root zone soil moisture, were sourced from NASA's Global Land Data Assimilation System Version 2.2 (GLDAS–2.2, https://disc.gsfc.nasa.gov/datasets/GLDAS_CLSM025_DA1_D_2.2/summary?keywords=GLDAS). To ensure spatial consistency with the NDVI data, all climate variables, including air temperature, precipitation, and the SPEI‐6 index, and hydrothermal condition variables were interpolated to a 250‐meter spatial resolution using the kriging interpolation method in ArcGIS.

**TABLE 1:** Datasets used in this study.

Datasets	Resolution	Year	Data source
MODIS NDVI	250 m × 250 m	2001–2021	https://appeears.earthdatacloud.nasa.gov/
CMA TMP	Stations	2001–2021	http://data.cma.cn/
CMA PRE	Stations	2001–2021	http://data.cma.cn/
GLDA SR	0.25° × 0.25°	2001–2021	https://disc.gsfc.nasa.gov/datasets/
GLDA SM	0.25° × 0.25°	2001–2021	https://disc.gsfc.nasa.gov/datasets/
GLDA RM	0.25° × 0.25°	2001–2021	https://disc.gsfc.nasa.gov/datasets/
DEM	90 m × 90 m		https://www.gscloud.cn
Vegetation types		2001	http://www.ncdc.ac.cn/
Boundary Karst			https://geocloud.cgs.gov.cn/

To facilitate the extraction and projection of Terra‐MOD13Q1 version 6.1 NDVI data for Guizhou Province, we employed AppEEARS, an online application developed by the Land Processes Distributed Active Archive Center (LP DAAC, https://appeears.earthdatacloud.nasa.gov/). This platform streamlines the process of regional data acquisition, which is essential for the present study. The retrieved NDVI dataset features a spatial resolution of 250 m and a temporal resolution of 16 days, spanning the period from January 1, 2001, to December 31, 2021. The data were subsequently preprocessed using ArcGIS 10.5. To minimize the effects of cloud contamination, the Maximum Value Composite (MVC) method was applied to calculate the monthly NDVI values. Thereafter, seasonal‐scale NDVI metrics for the study area were derived using the Image Analysis Statistics tool within ArcGIS 10.5.

The study also utilized datasets sourced from the National Glacial Tundra Desert Science Data Center (http://www.ncdc.ac.cn/), a part of the China Science and Technology Resource Sharing Service Platform. The dataset provides a comprehensive classification of Chinese vegetation types at a scale of 1:1,000,000. Given the significant impact of human activities on cultivated lands, such as agricultural farmlands, and the notably low representation of broadleaf and needleleaf mixed forests in Guizhou Province within the provided datasets, these categories were excluded from the analysis. This exclusion was necessary to avoid confounding the effects of human‐modified ecosystems when assessing the drought impacts on natural vegetation. Consequently, the focus of the study was on five primary natural vegetation types: broadleaf forests (BDF, 3.32%), needleleaf forests (NDF, 12.50%), scrublands (SCR, 31.48%), meadows (MDW, 1.80%), and grasslands (GRA, 15.62%). BDF are sparsely distributed, while SCR cover extensive areas. NDF are predominantly concentrated in the central‐eastern region, whereas GRA are most concentrated in the southwestern, southeastern, and northeastern regions. MDW are primarily located in the northwestern region, characterized by higher elevations.

In addition to the vegetation data, the analytical framework incorporated topographic and geomorphologic information, primarily consisting of DEM data and karst landscape distribution maps. The specific characteristics of all datasets utilized in this study are detailed in Table 1. Data processing was carried out using a combination of analytical tools, including R software, ArcGIS version 10.5, and Microsoft Excel, ensuring a robust and integrated approach to achieving the study's objectives.

### 2.3 Identification of Extreme Drought Events

The SPEI quantifies anomalies in the hydrologic balance to monitor drought conditions (Vicente‐Serrano et al. 2010, 2013). Previous research has demonstrated that the 6‐month SPEI (SPEI‐6) exhibits a stronger correlation with vegetation greenness compared to other timescales (Deng et al. 2021; Liu, Shan, et al. 2023). Accordingly, this study employed the regional average SPEI–6 and the run theory approach to identify drought events in Guizhou Province, characterize their spatial and temporal dynamics, and evaluate their impacts on vegetation. The methodology involved the following steps: (1) Considering the limited effect of mild drought on vegetation, a drought threshold of SPEI–6 ≤ −1.0 was established, whereby SPEI values below this threshold are indicative of drought conditions; (2) Drought events were defined as a period lasting longer than 1 month, with two droughts separated by < 1 month being merged into a single event. The duration of each drought event was determined as the interval from its onset to termination. Drought severity was classified according to the National Meteorological Drought Rating Criteria (see Table 2); (3) Seasons were delineated based on meteorological criteria as follows: spring (March–May), summer (June–August), autumn (September–November), and winter (December–February). Using these definitions, the seasonal average SPEI–6 index was calculated for Guizhou Province to quantify the seasonal characteristics of drought events.

**TABLE 2:** SPEI drought classification standard.

Drought category	SPEI value range
Mild drought	−1.0 < SPEI ≤ −0.5
Moderate drought	−1.5 < SPEI ≤ −1.0
Severe drought	−2.0 < SPEI ≤ −1.5
Extreme drought	SPEI ≤ −2.0

### 2.4 Quantifying Vegetation Response to Drought and Corresponding Hydrothermal Anomalies

To comprehensively evaluate the impact of drought events on vegetation growth and elucidate the underlying mechanisms, a comparative analysis was conducted using monthly NDVI and climate variables expressed as anomaly indices. Multiyear monthly average NDVI and hydrothermal condition data from 2001 to 2021 were employed as baseline values, with drought months excluded from baseline calculation in accordance with previous studies (Wolf et al. 2016; Wang, Ding, et al. 2021). Baseline values were determined based on the long‐term mean and the SPEI–6 index. Subsequently, anomalies in vegetation greenness and hydrothermal conditions were quantified relative to the long‐term mean using an anomaly index, a critical quantitative metric. Prior research has demonstrated the applicability of the SPEI–6 in conjunction with monthly NDVI (Liu, Shan, et al. 2023). Following the methodology of Tian et al.'s (2019), anomalies were classified as either positive or negative. Specifically, NDVI anomalies were calculated as the difference between observed monthly or seasonal NDVI values during drought events and the corresponding baseline values at the same temporal scale. Negative NDVI anomalies indicate a reduction in vegetation growth potentially attributable to drought stress, whereas positive anomalies suggest that drought conditions may have inadvertently promoted vegetation growth (Zhao et al. 2020; Wang, Ding, et al. 2021). The anomaly index was similarly applied to hydrothermal condition variables to assess their deviations from baseline conditions.

## 3 Results

### 3.1 Identification of Drought Events

#### 3.1.1 Temporal Evolution of Drought Events

**FIGURE 2:**
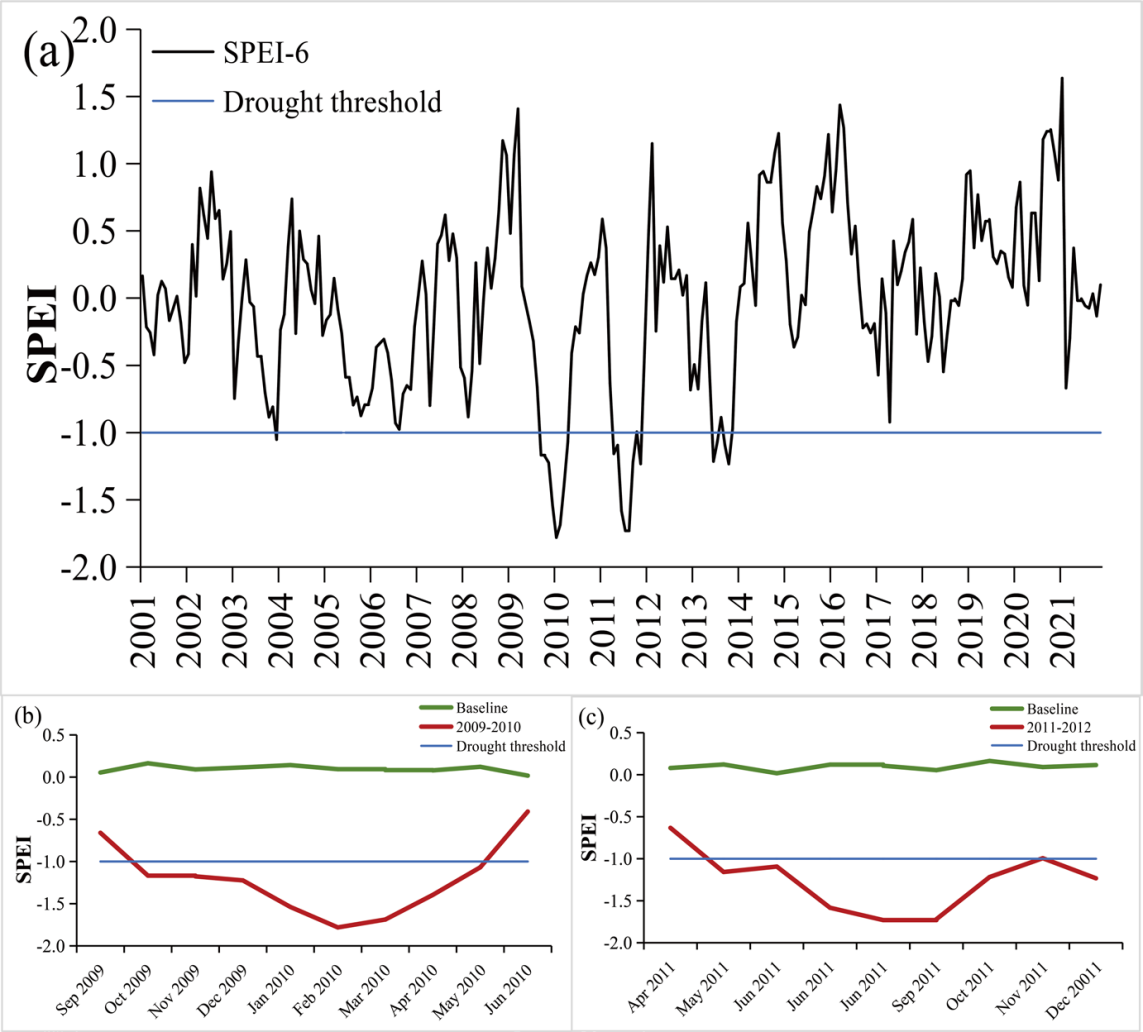
Monthly SPEI–6 time series for Guizhou Province: (a) full record (2001–2021), (b) 2009–2010 episode, and (c) 2011 episode. In (b) and (c), the green line represents the 2001–2021 monthly average baseline, the red line shows the SPEI variation during drought events, and the shaded area indicates drought duration.

As shown in Figure 2, our analysis identified drought events with varying severity in 2004, 2009–2010, 2011, and 2013. Notably, the droughts during 2009–2010 and 2011 were the most severe and prolonged, thereby serving as representative cases for in‐depth analysis in this study. With regard to the detailed temporal dynamics of these two drought events, the onset and cessation months were determined for each period. As shown in Figure 2b,c, relative to baseline conditions, SPEI values across Guizhou Province exhibited significant declines during these events, indicating pronounced drought conditions. The 2009–2010 drought commenced in October 2009 and persisted until May 2010, lasting approximately 8 months. The drought intensity intensified to severe levels from January to March 2010, peaking in February with SPEI values of −1.54, −1.78, and −1.69 for January, February, and March, respectively (Figure 2b). Similarly, the 2011 drought event began in May 2011, peaked between August and September, and concluded in December 2011, also spanning 8 months. During this period, drought severity reached severe levels in July, August, and September, with SPEI values of −1.58, −1.73, and −1.72, respectively (Figure 2c). Although several studies at the broader Southwest China regional scale have documented the spatiotemporal characteristics and vegetation responses associated with these drought events (Wang, Ding, et al. 2021; Zhou, Luo, et al. 2018; Dong et al. 2022), the heterogeneous nature of the region renders the drought dynamics with Guizhou Province distinct. Consequently, the spatiotemporal features of droughts in Guizhou exhibit unique attributes that differentiate them from regional‐level findings for Southwest China.

#### 3.1.2 Spatial Characteristics of the 2009–2010 and 2011 Drought

**FIGURE 3:**
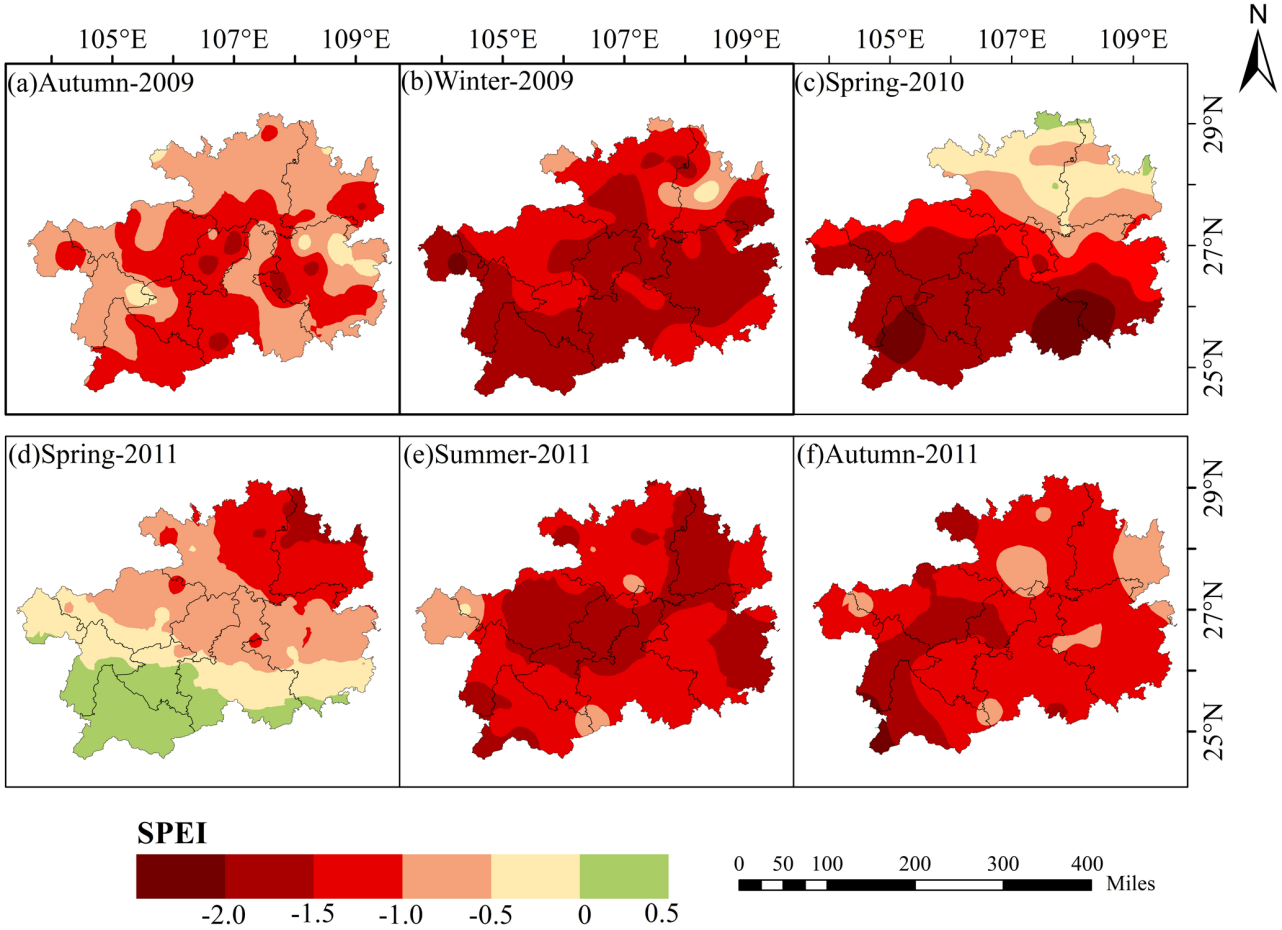
Seasonal spatiotemporal distribution of SPEI–6 during two major drought events in Guizhou Province: (a–c) from autumn 2009 to spring 2010, and (d–f) from spring to autumn 2011.

The spatial distribution of drought serves as a critical indicator of moisture deficits across geographic regions during drought events. In the case of the 2009–2010 and 2011 droughts in Guizhou Province, the seasonal‐scale characteristics of the SPEI–6, as depicted in Figure 3, reveal distinct spatial patterns. The 2009–2010 drought unfolded over three consecutive seasons: autumn, winter, and spring. During the initial phase in autumn, drought conditions began to emerge across parts of southern, central, and southeastern Guizhou Province. Notably, severe drought was observed in areas including Qiannan, Guiyang, and southeastern regions of the province. By the winter of 2009, drought had affected nearly the entire province, with the exception of localized areas in Tongren and Zunyi. Extremely severe drought conditions were widespread, particularly across the southwestern, southern, central, and southeastern regions. Among the hardest‐hit areas was Weining County, which experienced exceptionally acute drought impacts. By the spring of 2010, drought conditions had retreated southwestward from the northeast part of the province. Nevertheless, severe to exceptional drought persisted across substantial areas in the western, southern, and southeastern regions.

In contrast to the 2009–2010 drought, the 2011 drought in Guizhou Province demonstrated a distinct temporal and spatial pattern. This drought unfolded across three consecutive seasons: spring, summer, and autumn. It initially emerged in the northeastern part of the province, while the southwestern regions retained relatively stable moisture conditions. As the drought progressed, its intensity escalated during the summer months, eventually spreading across most of the province. While the majority of areas experienced moderate drought conditions, severe drought was observed in parts of the northeast, central, northwest, and southeast regions. By autumn, the areas affected by severe drought had shifted westward and northwestward. Despite this spatial transition, the drought did not subside immediately; some locations, particularly in Xingyi, continued to experience exceptionally severe drought conditions.

### 3.2 Response of Vegetation Greenness to the Two Drought Events

#### 3.2.1 Temporospatial Variations of Vegetation Response

**FIGURE 4:**
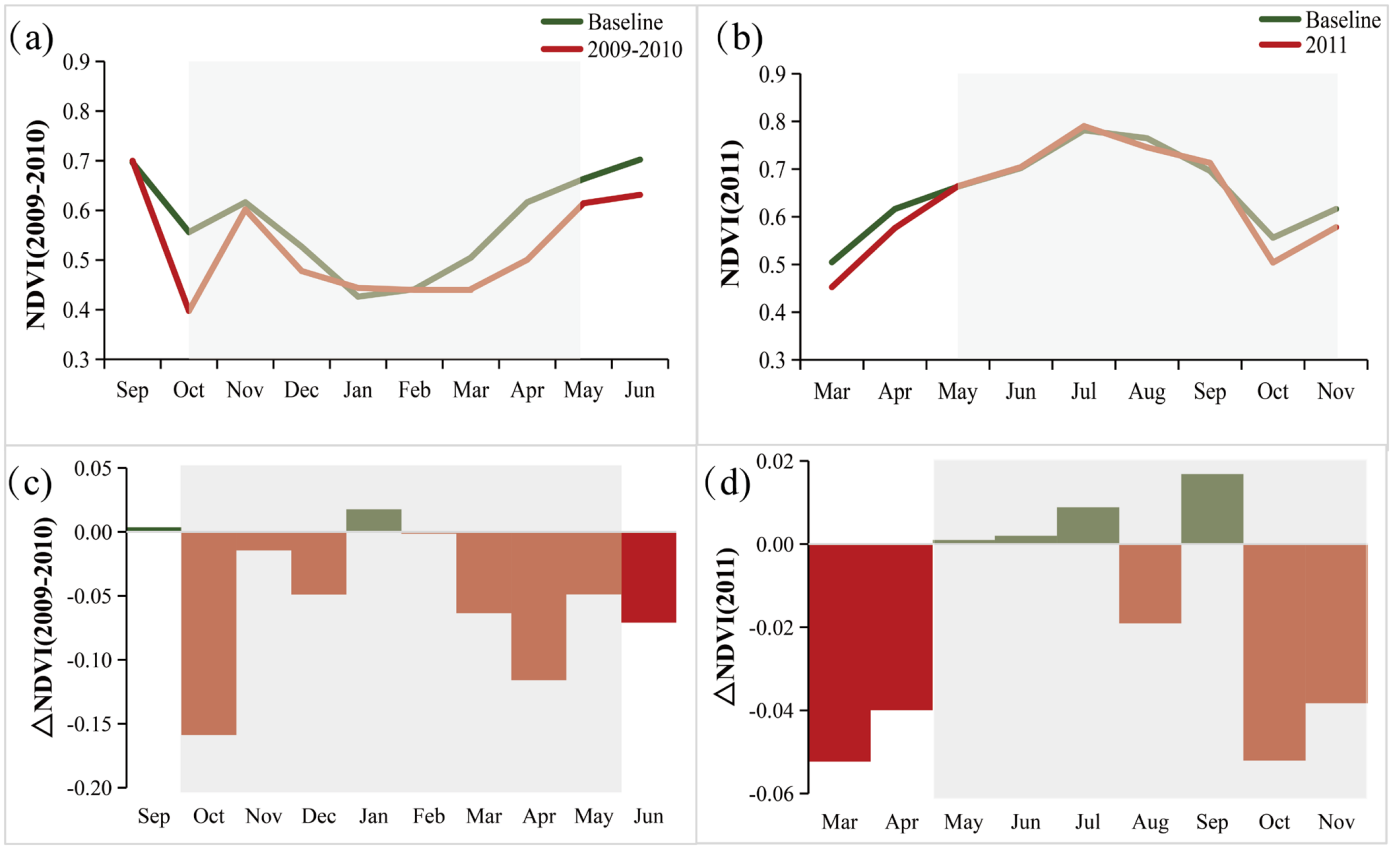
Temporal dynamics of NDVI in Guizhou Province during two drought events, relative to the 2001–2021 monthly mean baseline. (a, b) NDVI time series for the 2009–2010 and 2011 droughts, respectively; (c, d) corresponding deviation from the baseline. Shaded areas denote drought duration.

Figure 4 exhibits the temporal dynamics of the NDVI and its response to drought conditions in Guizhou Province. Variations in the baseline NDVI closely aligned with the natural vegetation growth cycle, with values beginning to rise from mid‐January to June, peaking in July, and subsequently declining to their lowest levels during the nongrowing season. During the 2009–2010 drought event, the NDVI exhibited a consistent negative anomaly relative to the baseline values, with an average decrease of −0.05 over the drought period spanning from October 2009 to May 2010. These anomalies were particularly pronounced during autumn (−0.06), winter (−0.01), and spring (−0.08). The most significant declines were recorded in October and April, with NDVI values decreasing by −0.16 and −0.12, respectively, corresponding to reductions of 28.54% and 18.40% compared to the baseline. Furthermore, the analysis revealed a temporal lag in the vegetation NDVI response to drought conditions. Under normal circumstances, NDVI values typically reached their minimum in January, followed by a gradual recovery as vegetation growth resumed. However, during the 2009–2010 drought, the NDVI minimum was delayed until March, indicating a lag of approximately 2 months in the recovery of vegetation activity. This delayed response underscores the prolonged impact of drought stress on vegetation growth dynamics in the region.

In contrast, the 2011 drought event exerted comparatively weaker inhibitory effects on vegetation growth, with certain regions even experiencing enhanced NDVI values under drought conditions. Prior to the onset of the drought, NDVI values declined by 10.36% and 6.48% relative to baseline levels in March and April 2011, respectively. However, despite the development and intensification of drought conditions during late spring and summer, NDVI values unexpectedly increased by 0.15%, 0.29%, 1.13%, and 2.42% in May, June, July, and September 2011, respectively, compared to baseline values. This observation challenges the conventional assumption that vegetation growth is uniformly suppressed during drought events. Following this period of relative resilience, NDVI values declined by 9.36% and 6.21% in October and November 2011, respectively, coinciding with the gradual alleviation of drought conditions. These findings underscore the complex and nonlinear relationship between drought stress and vegetation response, suggesting that the impacts of drought on vegetation dynamics may vary both spatially and temporally, depending on local environmental conditions and ecosystem adaptability.

**FIGURE 5:**
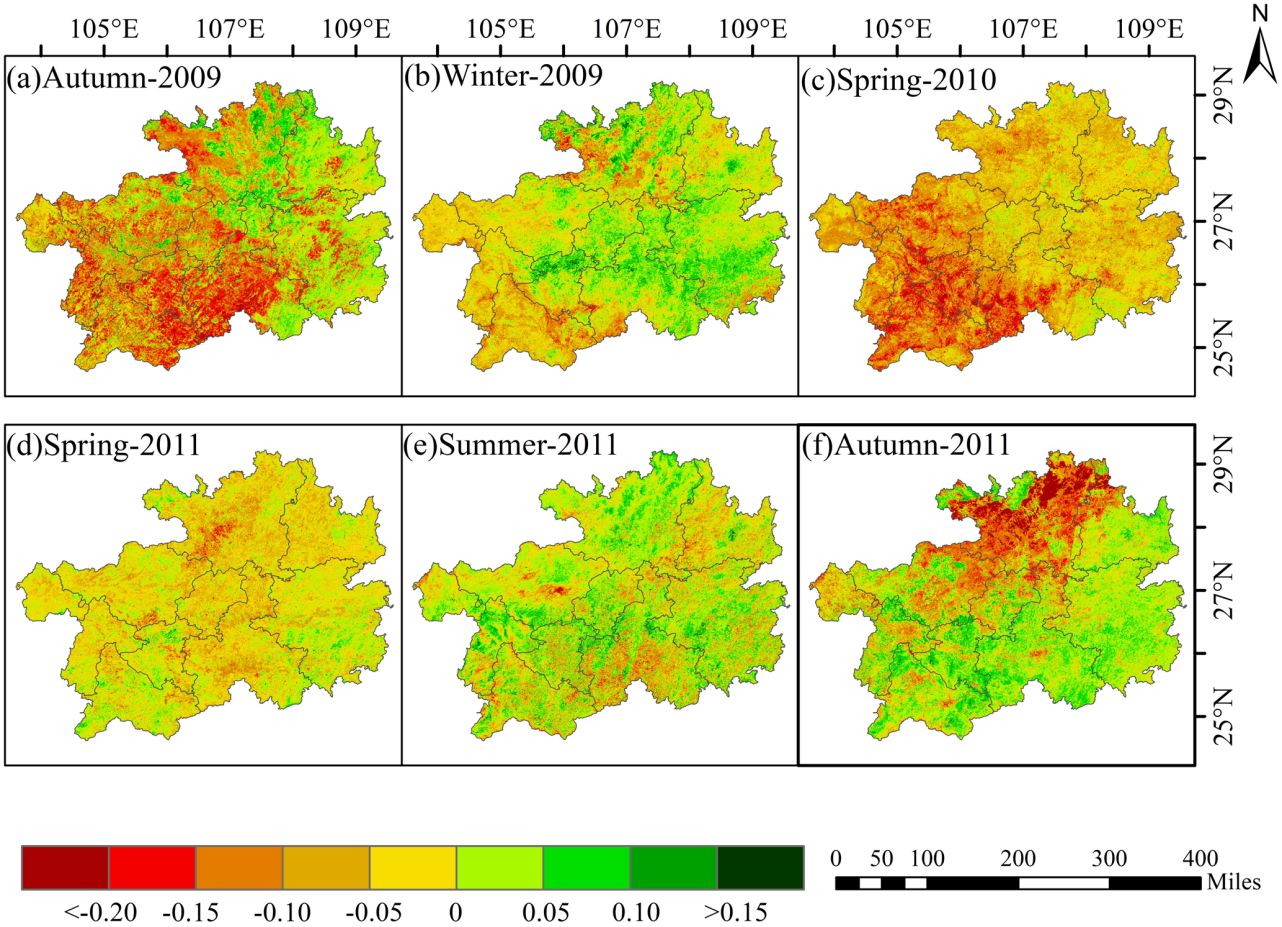
Spatial patterns of seasonal NDVI anomalies during two drought events in Guizhou Province: (a–c) fall–winter–spring 2009–2010, and (d–f) spring–summer–fall 2011 (relative to baseline conditions).

The protracted drought conditions during the 2009–2010 period exerted a pronounced impact on seasonal vegetation growth, characterized by marked spatial variability in vegetation response to water stress (see Figure 5). In autumn 2009, vegetation greenness exhibited varying degrees of degradation, with the most pronounced negative NDVI anomalies observed across central, southern, and western Guizhou Province. In contrast, some areas in the easter and northern parts of the province displayed anomalous positive NDVI response. Although the severity of the drought peaked during the winter of 2009, the extent of NDVI degradation appeared to be somewhat moderated compared to the preceding season. The most critical phase occurred in the spring of 2010, which negative NDVI anomalies were recorded across the entire province. The southwestern region, in particular, experienced the most substrantial declines, indicating a severe suppression of vegetation growth.

In the spring of 2011, NDVI anomalies across the majority of Guizhou Province ranged from −0.05 to 0, indicating generally subdued vegetation activity. During the summer season, positive NDVI anomalies were widespread throughout the province, with the exception of a few localized areas in the southern and northeastern regions where anomalies remained negative. The autumn season witnessed a distinct spatial distribution characterized by a juxtaposition of positive and negative anomalies. Negative anomalies were primarily concentrated in the northern regions of Guizhou Province, with values falling below −0.15 in certain areas. In contrast, positive anomalies were more prominent and exhibited greater magnitudes in the central, southern, eastern, and southwestern regions of Guizhou Province.

#### 3.2.2 Response of Different Types of Vegetation

**FIGURE 6:**
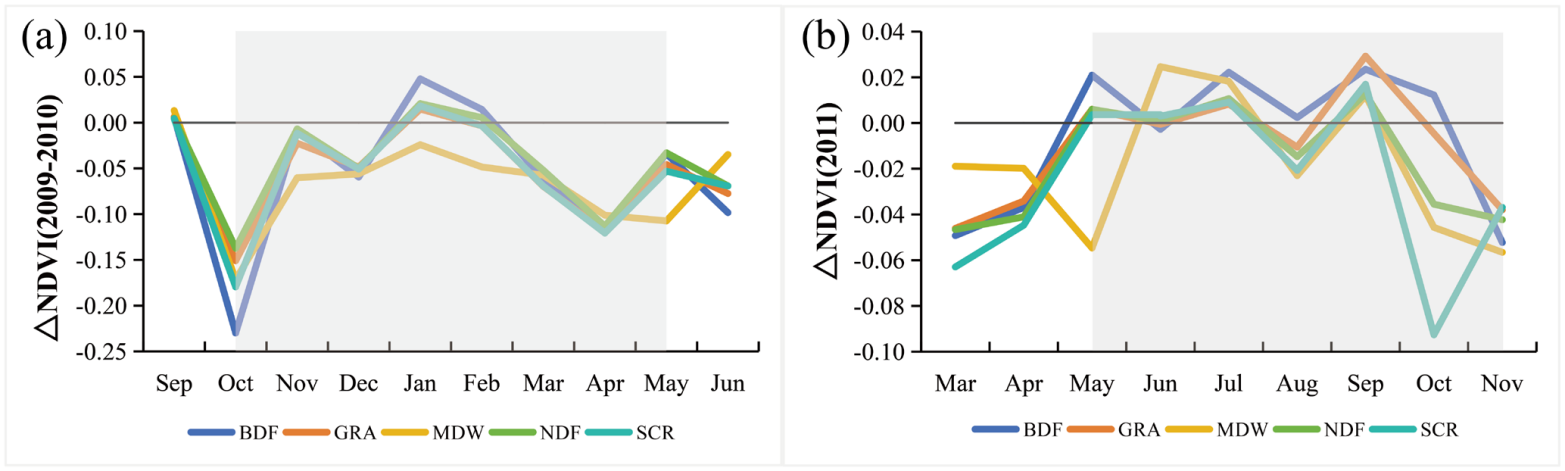
NDVI response to extreme drought across different vegetation types (broadleaf/needleleaf forests, grasslands, scrublands, meadows) in Guizhou Province: (a) 2009–2010 event; (b) 2011 event.

Figure 6 presents a comparative analysis of vegetation greenness across various vegetation types during the two drought episodes. The results indicated that the monthly NDVI for different vegetation types generally mirrored the overall NDVI variations shown in Figure 4, albeit with certain nuanced differences. Meadows, predominantly located in the higher‐elevation western regions, exhibited the most pronounced negative anomaly, with a reduction of −0.08 (16.21%) relative to baseline values. Scrublands, broadleaf forests, and grasslands exhibited similar patterns of declines, with NDVI decreases of −0.0588 (10.91%), −0.0559 (9.84%), and −0.0556 (9.56%), respectively. In contrast, needleleaf forests experienced the smallest reduction, with a decline of −0.0457 (8.12%). These findings suggest that while all vegetation types were affected by the drought, the severity of the impact varied considerably, with meadows proving to be the most sensitive to drought conditions among the vegetation types analyzed.

Despite comparable durations and intensities, the spatial extent of NDVI anomalies during the 2011 drought was notably smaller than that observed during the 2009–2010 event. Furthermore, the magnitude of NDVI anomalies varied considerably across different vegetation types during these two drought episodes. Notably, broadleaf forests exhibited a positive anomaly in mean NDVI, with an increase of 0.0037 (0.5%), maintaining a relatively high level of greenness even during the peak of summer, despite the drought reaching its most severe phase. In contrast, all other vegetation types displayed negative anomalies in mean NDVI. Among these, scrublands experienced the most pronounced negative anomaly (−0.025), along with considerable variability, suggesting a high sensitivity to drought stress relative to other vegetation types.

In summary, the findings highlight that different vegetation types exhibit distinct responses to seasonal drought conditions. Winter–spring drought was found to significantly suppress growth across all vegetation types, with meadows showing the highest sensitivity to water stress, while needleleaf forests demonstrated the greatest drought resistance. The summer drought of 2011, occurring during the active growing season, elicited varied responses in vegetation NDVI, particularly in the early to middle stages of the drought. Broadleaf forests, in particular, maintained the highest levels of greenness, likely due to their well‐developed deep root systems that facilitated optimal utilization of deep groundwater reserves. In contrast, meadows exhibited greater sensitivity to soil moisture fluctuations, as reflected in their more pronounced NDVI variations. As the drought intensity intensified, all vegetation types experienced differential declines in growth during the later stage of the drought, particularly in October. Among these, scrublands showed the most significant degradation in photosynthetic capacity, highlighting their heightened vulnerability to prolonged drought conditions.

#### 3.2.3 Vegetation Responses to Extreme Drought Across Geomorphic Landforms

**FIGURE 7:**
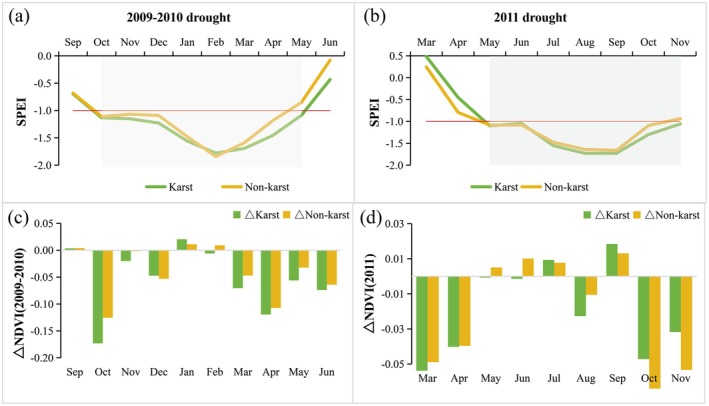
Comparison of drought severity (a, b) and NDVI deviation (c, d) between karst and non‐karst regions in Guizhou Province for the 2009–2010 (a, c) and 2011 (b, d) drought events.

To examine the differential response of vegetation greenness to extreme droughts in karst and non‐karst landforms, Figure 7 presents the drought conditions and NDVI anomalies for each landscape. The results indicate that the karst regions experienced more severe drought conditions compared to the non‐karst regions during both the 2009–2010 and 2011 drought events (Figure 7a,b). During the 2009–2010 drought event, the NDVI in the non‐karst region exhibited weaker negative anomalies relative to the karst regions (Figure 7c). In contrast, during the 2011 drought event, the disparity in drought intensity between the two geomorphological types was less pronounced (Figure 7d). Notably, there was no clear manifestation of the typical response pattern, where karst vegetation tends to show greater growth suppression. Interestingly, during the early to middle phases of the drought (May–July and September), vegetation growth in both regions remained largely unaffected and even displayed enhanced photosynthetic activity, as indicated by increased greenness. Of particular note, non‐karst regions exhibited superior growth performance compared to karst areas from May to June. However, this pattern reversed during the late growing season (October–November), when non‐karst vegetation experienced more substantial drought‐induced suppression. In conclusion, a comparative analysis of the two extreme drought events reveals that karst regions consistently endured higher drought severity than non‐karst areas. This hydrological stress gradient resulted in significantly stronger drought‐induced growth inhibition in karst vegetation compared to non‐karst ecosystems.

**FIGURE 8:**
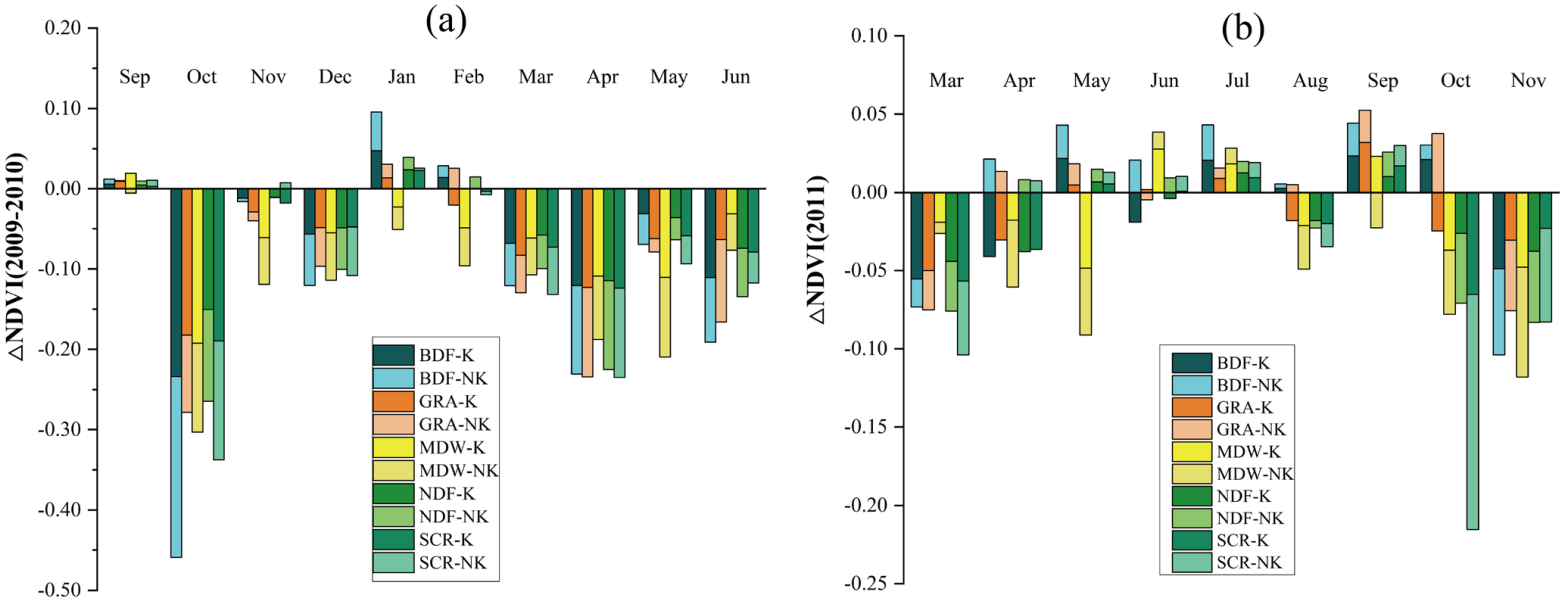
Differential vegetation response to extreme drought across karst (K) and non‐karst (NK) zones by vegetation type during (a) 2009–2010 and (b) 2011 (stacked bar chart).

To further investigate variations in vegetation responses to drought across different vegetation types in karst and non‐karst landforms, an additional analysis was conducted. As shown in Figure 8a, during the 2009–2010 drought event, negative NDVI anomalies were generally more pronounced for most vegetation types within the karst regions compared to their counterparts in non‐karst areas. However, this trend was less apparent during the 2011 drought. While the negative NDVI anomalies were somewhat greater for most vegetation types in the karst regions during March, April, and August, the opposite pattern emerged in October and November. Specifically, vegetation in the non‐karst areas exhibited more substantial declines than in karst regions, with scrublands in the non‐karst landscape experiencing the most pronounced decrease in October (−0.1583). Additionally, minimal differences were observed between the two landforms in the months when NDVI showed slight increases (Figure 8b).

### 3.3 Possible Mechanisms

#### 3.3.1 Temporal Variations of Hydrothermal Conditions

**FIGURE 9:**
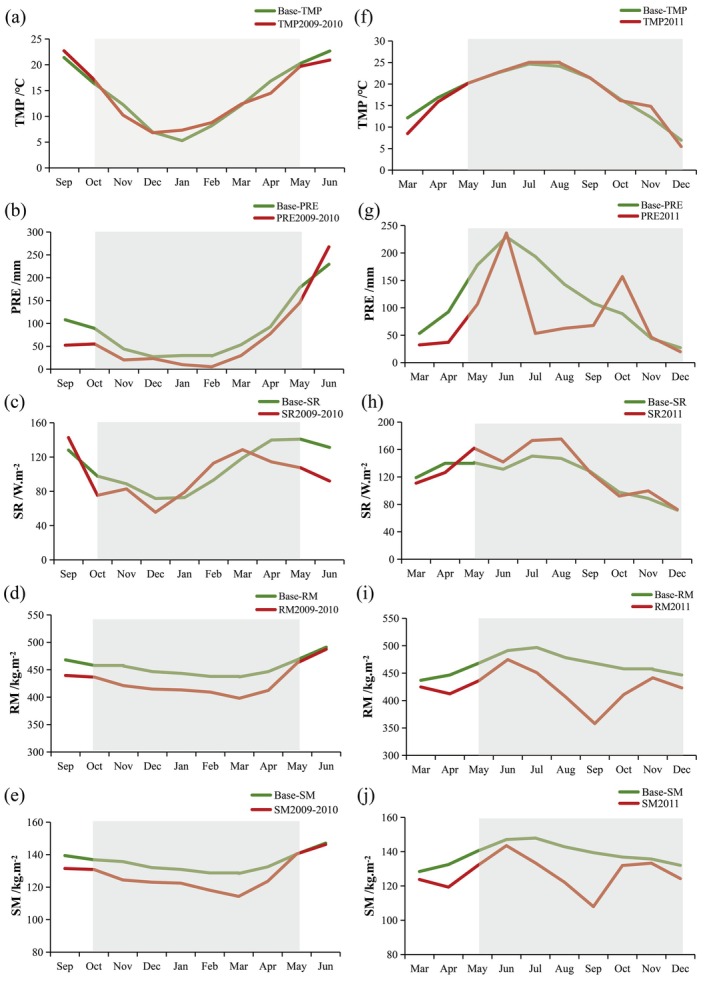
Anomalies in key hydrothermal variables during the 2009–2010 and 2011 droughts in Guizhou Province, relative to the 2001–2021 monthly mean baseline. Variables shown are: (a, f) temperature (TMP), (b, g) precipitation (PRE), (c, h) net shortwave radiation (SR), (d, i) root‐zone soil moisture (RM), and (e, j) soil moisture (SM). The green line indicates the baseline level, and the red line shows drought‐period anomalies.

Although the intensity and duration of the drought events in 2009–2010 and 2011 were similarly severe, significant differences in climatic conditions and seasonal dynamics were observed between the two periods. Correspondingly, the vegetation greenness response to these droughts exhibited distinct patterns. These discrepancies can be attributed to the differing hydrothermal conditions that prevailed during each drought episode. As such, it was necessary to investigate the underlying mechanisms responsible for these observed differences. Figure 9 shows the regional mean variations in key hydrothermal factors, including temperature, precipitation, net shortwave radiation flux, root‐zone soil moisture, and surface soil moisture content, during both the 2009–2010 and 2011 drought events.

Distinct negative anomalies in the SPEI–6, below −1.0 relative to baseline condition, were observed from October 2009 to May 2010 (Figure 2b). The onset of the 2009–2010 drought was marked by a notable reduction in precipitation from September to December 2009. Specifically, precipitation levels decreased by −34.2 mm (38.3%), −23.9 mm (54.2%), −4.0 mm (14.7%) in comparison to baseline values during September, October, and December, respectively. Simultaneously, temperatures and shortwave radiation experienced a cooler phase, particularly between November and December 2009. The severity of the drought intensified from January to March 2010, driven by a combination of persistent precipitation deficits, abrupt warming, and elevated shortwave radiation levels. These conditions persisted through May 2010, after which the drought began to subside, largely due to a sharp increase in precipitation and a subsequent drop in temperatures during June 2010. The prolonged lack of precipitation, coupled with rising temperatures and intensified shortwave radiation, significantly exacerbated soil evaporation rates, leading to a subsequent depletion of soil moisture. These adverse hydrothermal conditions, characterized by extreme drought and reduced soil moisture, contributed to a sustained decline in vegetation greenness throughout the 2009–2010 drought period.

The 2011 drought, as depicted in Figure 2c, was characterized by distinct negative anomalies in the SPEI–6 index from May to December of that year. Prior to the onset of the drought, there was a marked decrease in precipitation, with deficits of 39.4% and 59.9% (−30.0 and −55.5 mm, respectively) in March and April 2011 relative to the baseline condition. This precipitation shortfall, coupled with temperature decreases of −3.65°C and −0.98°C, created dry and cold climatic conditions. Although the reduced temperatures might have theoretically mitigated evapotranspiration, they also impeded the vegetation's ability to accumulate the necessary temperature for germination, thereby hindering the growth and development of vegetation in the spring (Figure 4b,d). With persistent precipitation deficits, the drought formally began in May and reached its peak between August and September 2011, marked by substantial reductions in precipitation of 72.5% (−140.2 mm), 56.2% (−80.3 mm), and 37.2% (−40.3 mm) from July to September, respectively. In contrast to the detrimental effects of the 2009–2010 drought event on vegetation growth, the early and intermediate stages of the 2011 drought paradoxically supported vegetation growth, with a slight increase in greenness. This counterintuitive phenomenon can be attributed to a sudden increase in precipitation in June, which alleviated soil moisture deficits, coupled with an increase in net shortwave radiative flux that promoted photosynthesis activity in vegetation. However, as the drought persisted, significant reductions in both surface and root‐zone soil moisture were observed, with a decrease of 23.5% and 22.6%, respectively, by September. The depletion of water resources ultimately rendered the environment incapable of sustaining plant growth, resulting in premature leaf senescence and yellowing in certain vegetation types. Consequently, the greenness of vegetation experienced a marked decline during October and November 2011 (Figures 4b,d and 9i,j).

#### 3.3.2 Hydrothermal Conditions of Different Types of Vegetation

**FIGURE 10:**
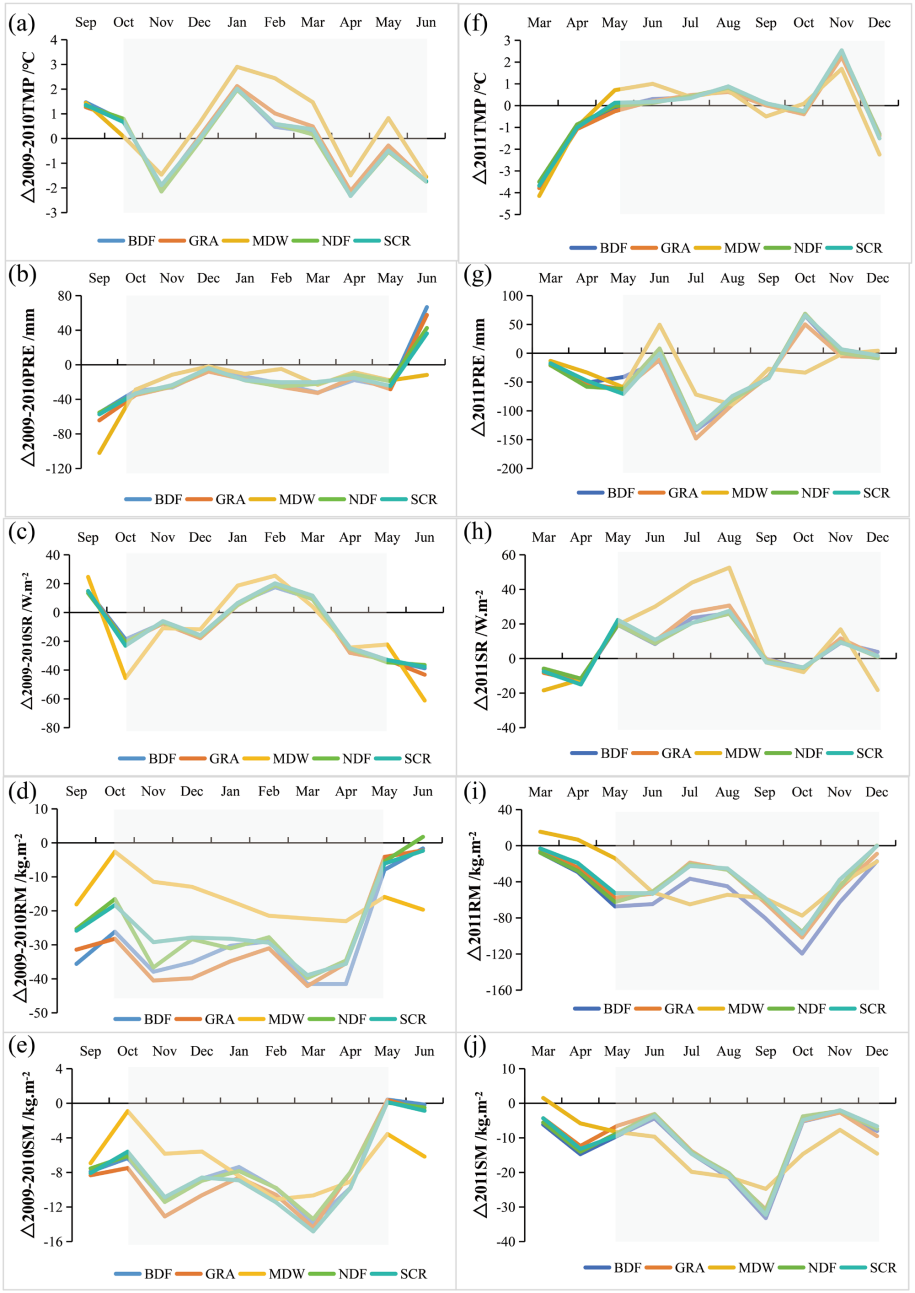
Same as Figure 9, but for the hydrothermal conditions deviation across different vegetation types during the 2009–2010 and 2011 drought events.

Figure 10 presents the hydrothermal anomalies for various vegetation types relative to the baseline conditions during the 2009–2010 and 2011 droughts. In general, woody vegetation exhibits greater resistance to drought than herbaceous vegetation, primarily due to its deeper root system, which enables more efficient extraction of groundwater (Liu, Chen, et al. 2021; Jiang et al. 2023). During the 2009–2010 drought, meadow ecosystems experienced the most significant temperature increase among all vegetation types (Figure 10a). Their shallow‐rooted structure, with roots concentrated primarily in the topsoil, made them particularly vulnerable to thermal stress exacerbated by precipitation deficits. As a result, meadows exhibited the most pronounced decline in vegetation greenness (Figure 6a), with the suppression effects significantly surpassing those observed in other vegetation types. These findings align with established ecological principles regarding plant‐water relations. A comparative analysis further reveals that needleleaf forests demonstrate superior drought tolerance relative to other vegetation types. This enhanced resilience is attributed to their xeromorphic adaptations, such as waxy cuticles, reduced leaf area, and sunken stomata, in addition to their resource‐conserving strategies. These structural and physiological traits allow conifers to sustain photosynthetic activity with minimal reductions in greenness, even under comparable levels of hydrothermal stress.

Our findings indicate that grasslands exhibit greater drought resistance than broadleaf forests. This differential response may be attributed to the fine‐scale landscape heterogeneity characteristic of Guizhou's karst terrain. Such heterogeneity fosters a patchy distribution of grassland vegetation, leading to the formation of localized microenvironments that are more resilient under drought conditions. In contrast, forests typically possess more developed root systems, which allow for deeper soil water extraction, thereby supporting growth and survival during periods of water scarcity (Liu, Chen, et al. 2021). Consequently, forests with high species richness and complex structural composition tend to demonstrate greater ecosystem stability and enhanced drought resistance. However, forest responses to drought are influenced by a combination of climatic factors—such as temperature and precipitation—and intrinsic forest traits, including species composition, stand age, and canopy height (Choat et al. 2005; Xu et al. 2022). Notably, evergreen species, which require higher water availability to sustain foliar growth, are particularly susceptible to drought stress (Choat et al. 2005). Emerging evidence suggests that canopy height is a key functional trait that significantly modulates forest drought responses (Chen, Zhao, et al. 2024; Liu, Chen, et al. 2021; Xu et al. 2022). This relationship is linked to the physiological connection between canopy architecture and xylem morphology. Taller canopies are associated with longer xylem conduits, which facilitate the transport of water from the roots to the foliage during photosynthesis (Chen, Zhao, et al. 2024; Fu et al. 2012; Jiang et al. 2023; Liu, Chen, et al. 2021 ). However, taller forests also face greater hydraulic constraints, as longer water transport pathways and increased transpiration demands reduce hydraulic efficiency. These constraints heighten the risk of xylem cavitation and embolism, which can result in dieback and mortality (Fu et al. 2012; Chen, Zhao, et al. 2024; Liu, Chen, et al. 2021; Xu et al. 2025).

Grassland ecosystems, characterized by shallow root systems, are unable to access deep groundwater resources. However, these ecosystems employ efficient water‐use strategies that enhance their drought resistance. Such strategies include reduced stomatal aperture and suppressed transpiration in C4 grasses, as well as nocturnal physiological activity coupled with diurnal stomatal closure. Liu, Shan, et al. (2023) demonstrated that, in comparison to grasslands, forests in Southwest China exhibit significantly greater demands on both soil moisture and groundwater resources. Additionally, the relatively minor differences in hydrothermal conditions between grasslands and broadleaf forests in Guizhou Province during drought periods (Figure 10a–e) suggest that broadleaf forests become particularly vulnerable to xylem embolism when drought stress exceeds a critical threshold, and groundwater availability is insufficient to meet their physiological demands. As a result, broadleaf forests face considerably higher risks of browning compared to grassland ecosystems, leading to more severe drought impacts on forested areas than on grasslands.

During the 2011 drought event, all vegetation types were subjected to elevated temperatures, increased precipitation in June, heightened shortwave radiation, and reduced soil moisture during the early to middle phases of the drought (Figure 10f–j). A comparative analysis revealed that meadows exhibited a higher degree of hydrological sensitivity compared to other vegetation types. Specifically, meadows demonstrated the most pronounced enhancement in greenness (ΔNDVI) during June, responding strongly to the combined effects of elevated temperatures, increased precipitation, and intensified shortwave radiation. In contrast, while other vegetation types experienced relatively stable hydrothermal conditions, broadleaf forests showed the most significant greenness enhancement during the early to mid‐drought period. This response can be attributed to the forest's extensive root systems and their competitive strategies for resource acquisition, which enabled them to better capitalize on available water and solar energy.

## 4 Discussion

### 4.1 Differential Vegetation Responses to Droughts and Potential Mechanisms

This study, utilizing data from 83 national meteorological stations and MODIS NDVI observations, investigates the response of vegetation greenness to the consecutive drought events that occurred during the fall–winter–spring period of 2009–2010 and the spring–summer–fall period of 2011 in Guizhou Province. Consistent with the findings of Song et al.'s (2019) for Southwest China, the winter–spring drought of 2009–2010 significantly suppressed vegetation growth. However, the summer drought of 2011 did not exert a similarly severe inhibitory effect on vegetation during the initial and mid‐drought phases; rather, it appeared to promote vegetation growth to a certain extent. These results highlight seasonal differences in vegetation response to drought.

The present study also indicates that the drought in Guizhou Province persisted for 8 months, suggesting spatial and temporal heterogeneity in vegetation responses to drought at the regional scale. Furthermore, previous studies (Tang and Dubayah 2017; Liu, Sun, et al. 2023; Xue et al. 2024; Xue and Chen 2025) have demonstrated that precipitation and increased shortwave radiation can mitigate the negative effects of drought on vegetation to some degree. Through an analysis of hydrothermal conditions, this study found that the persistent precipitation deficits and high temperatures from 2009 to 2010 were critical factors in suppressing vegetation growth. The prolonged water deficit had a severe impact on the physiological growth processes of vegetation (Dong et al. 2022), impairing overall plant health and leading to a delayed recovery into the following spring. This delayed recovery, or hysteresis effect, hindered the vegetation's ability to rebound rapidly (Guo et al. 2023; Li et al. 2023), ultimately resulting in a substantial decline in vegetation NDVI values relative to baseline conditions.

In contrast to the 2009–2010 drought, which primarily occurred during the winter–spring period, the 2011 drought began in late spring, when vegetation had already developed some degree of drought tolerance. By this time, the vegetation was in an active growth phase, and the substantial precipitation in June, coupled with elevated shortwave radiation during the early to mid‐drought periods, helped mitigate the drought's suppressive effects on growth. However, a hysteresis effect was evident throughout this event. Specifically, while vegetation continued to grow during the peak of the drought in the summer, the most severe browning occurred in the late stages of the drought, when the intensity of the drought had subsided. This delayed response underscores the complex and multifaceted nature of vegetation's physiological adaptation to drought conditions.

### 4.2 Heterogeneous Response of Various Vegetation Types to Seasonal Drought

The study further elucidated the heterogeneous response of various vegetation types to seasonal drought, with observed variations primarily attributable to differences in hydrothermal conditions and the inherent physiological traits of each vegetation type. The results indicated that meadows exhibited the highest sensitivity to moisture deficits, while needleleaf forests demonstrated the greatest drought resistance during the winter and spring droughts. Conversely, broadleaf forests were more adversely affected by drought conditions than grasslands when sustained moisture deficit occurred. However, during the summer and fall droughts, groundwater reservoirs, accumulated prior to and during the early to mid‐drought phases, were sufficient to meet the growth demands of broadleaf forests, resulting in significant vegetation growth during these periods. In contrast, the shallow root systems of scrublands and meadow grasslands limited their ability to compete for deeper soil water with the deep‐rooted broadleaf and needleleaf forests. This disparity in root depth and water access further exacerbated the vulnerability of scrublands and meadows to drought conditions (Ding et al. 2020; Lin et al. 2023).

### 4.3 Differential Drought Responses in Karst and Non‐Karst Landforms

Although karst vegetation has developed physiological traits and growth strategies adapted to the unique challenges of its environment, the findings of this study indicate that karst landscapes are more susceptible to intensified drought severity, heightened vulnerability to drought stress, and more significant anomalies in vegetation greenness when compared to non‐karst regions. This disparity can be attributed to the inherent characteristics of karstic landforms, such as thinner soil profiles, reduced water storage capabilities, and increased rates of seepage, all of which exacerbate the effects of drought. As a result, karst areas experience widespread signs of dehydration, including browning, wilting, and vegetation mortality under conditions of severe water scarcity (Lu et al. 2023; Yang et al. 2024). In contrast, non‐karst regions, characterized by thicker soil layers and superior water retention properties, exhibit greater resilience to drought impacts, thereby mitigating the adverse effects on vegetation growth (Xu, Wu, et al. 2021; Xie et al. 2023).

### 4.4 Limitations and Prospects

This study provides valuable insights into the response of vegetation to drought events in Guizhou Province, shedding light on the underlying ecological mechanisms. However, several limitations must be acknowledged. Firstly, the temporal resolution of this study, which operates at a seasonal scale, is insufficient for capturing the finer details of drought progression and the corresponding vegetation response. A more granular temporal approach, such as monthly or pentad scales, would enable a more precise representation of the dynamics involved. Secondly, the study focuses solely on the drought periods, neglecting the subsequent stages of vegetation recovery. This omission restricts a comprehensive understanding of vegetation resilience, an essential component of the drought cycle. Future research should thus incorporate the assessment of the post‐drought recovery phase to offer a more holistic view of the ecological impact of drought. Lastly, while the study highlights the sensitivity of vegetation in karst regions to drought, it primarily concentrates on hydrothermal factors during drought periods. The scope of environmental variables considered is limited, and future studies should expand this by integrating additional ecophysiological factors, such as vapor pressure deficit, fraction of absorbed photosynthetically active radiation, and evapotranspiration. Moreover, employing advanced techniques like random forest modeling could help identify the key drivers of vegetation response to drought. Such a comprehensive approach would provide critical scientific support for addressing the increasing frequency of drought events in Guizhou Province, particularly under future climate change scenarios. Ultimately, this research can inform the development of evidence‐based ecological conservation strategies, drought mitigation measures, and disaster prevention protocols.

## 5 Conclusion

This study employed the SPEI and NDVI to investigate the spatial and temporal dynamics of drought events in Guizhou Province during the 2009–2010 and 2011 periods. The study further explored the differential response of vegetation across various vegetation types and geomorphological settings. The findings revealed that both drought events were classified as severe, with duration extending for eight consecutive months. The 2009–2010 drought was most pronounced during the winter season, with the southwestern region of Guizhou Province experiencing the most significant impacts. In contrast, the 2011 drought reached its peak intensity during the summer months, with the western part of Guizhou Province facing more severe conditions. The 2009–2010 drought had a more substantial inhibitory effect on vegetation growth, as evidenced by a notable reduction in NDVI during the spring. Among the five selected vegetation types examined, all exhibited negative NDVI anomalies, with meadows showing the most severe suppression, followed by scrublands, broadleaf forests, and grasslands, while needleleaf forests were the least affected. In contrast, the 2011 drought had a relatively milder impact, with certain vegetation types, notably broadleaf forests, exhibiting continued vegetative growth despite the prevailing drought conditions. The vegetation response to drought was also found to vary across different landscape types. Specifically, karstic landforms were more prone to greater drought severity compared to non‐karstic landforms, a trend mirrored by the more pronounced impact of drought on vegetation greenness in karst regions. Under conditions of extreme drought severity, changes in hydrothermal variables—particularly precipitation and shortwave radiation—played a significant role in shaping the response dynamics of vegetation across both vegetation types and landform types.

## Author Contributions

**Chuncan Meng:** conceptualization (equal), methodology (equal), visualization (equal), writing – original draft (equal), writing – review and editing (equal). **Yingqing Cen:** methodology (equal). **Xu Xue:** conceptualization (equal), methodology (equal), supervision (equal), writing – review and editing (equal).

## Funding

This work was supported by Guizhou Provincial Basic Research Program (Natural Science) (Grant No. QianKeHeJiChu–ZK[2024]YiBan025); National Natural Science Foundation of China (Grant No. 42465003 and Grant No. 41965004).

## Conflicts of Interest

The authors declare no conflicts of interest.

## Data Availability

The data that support the findings of this study are openly available at https://disc.gsfc.nasa.gov/datasets/GLDAS_CLSM025_DA1_D_2.2/summary?keywords=GLDAS and https://appeears.earthdatacloud.nasa.gov/.
